# Author Correction: Molecular bottlebrush prodrugs as mono- and triplex combination therapies for multiple myeloma

**DOI:** 10.1038/s41565-023-01345-y

**Published:** 2023-03-13

**Authors:** Alexandre Detappe, Hung V.-T. Nguyen, Yivan Jiang, Michael P. Agius, Wencong Wang, Clelia Mathieu, Nang K. Su, Samantha L. Kristufek, David J. Lundberg, Sachin Bhagchandani, Irene M. Ghobrial, P. Peter Ghoroghchian, Jeremiah A. Johnson

**Affiliations:** 1 Department of Medical Oncology, Dana-Farber Cancer Institute, Boston, MA USA; 2 Harvard Medical School, Boston, MA USA; 3 Institut de Cancérologie Strasbourg Europe, Strasbourg, France; 4 Department of Chemistry, Massachusetts Institute of Technology, Cambridge, MA USA; 5 Window Therapeutics, Boston, MA USA; 6 Department of Chemical Engineering, Massachusetts Institute of Technology, Cambridge, MA USA; 7 Koch Institute for Integrative Cancer Research, Massachusetts Institute of Technology, Cambridge, MA USA; 8 Broad Institute of MIT and Harvard, Cambridge, MA USA

**Keywords:** Drug delivery, Nanotechnology in cancer

Correction to: *Nature Nanotechnology* 10.1038/s41565-022-01310-1. Published online 26 January 2023.

In the version of this article originally published, a conversion error led to parts of the structures in Figure 1a not appearing. The figure has been corrected in the HTML and PDF versions of the article.

